# Cost-effectiveness of adding measurement of *Chlamydia trachomatis* infection and serology to trachoma prevalence surveys in Tanzania and Mozambique

**DOI:** 10.1371/journal.pntd.0013257

**Published:** 2025-07-21

**Authors:** Emily C. Decker, Molly W. Adams, William E. Oswald, Rebecca M. Flueckiger, Jeremiah M. Ngondi, Mawo Fall, Ezgi E. Yilmaz, Lisa C. Brooks, George Kabona, Henis Mior Sitoe, Mabula Kasubi, Tamimo Momade, Thomas M. Lietman, Thuy Doan, Rachel D. Stelmach

**Affiliations:** 1 Duke University, Durham, North Carolina, United States of America; 2 RTI International, International Development Group, Washington, Columbia, United States of America; 3 RTI International, International Development Group, Maputo, Maputo, Mozambique; 4 Cornell University, Dyson School of Applied Economics and Management, Ithaca, New York, United States of America; 5 Ministry of Health Community Development Gender Elderly and Children, Dodoma, Dodoma, Tanzania; 6 Ministério da Saúde, Maputo, Maputo, Mozambique; 7 Muhimbili University of Health and Allied Sciences, Department of Microbiology, Dar es Salaam, Tanzania; 8 The Francis I. Proctor Foundation, University of California, San Francisco, San Francisco, California, United States of America; Medical University of Vienna, AUSTRIA

## Abstract

**Background:**

Accurate methods to measure trachoma prevalence are critical to monitor progress and guide mass drug administration as countries near elimination. Currently, countries conduct trachoma prevalence surveys via clinical examination using the simplified trachoma grading system. Grading can have reduced accuracy in low prevalence settings, potentially resulting in errors. Adding ocular swabbing and *Chlamydia trachomatis* (*Ct*) infection testing and dried blood spot (DBS) collection and testing can be more sensitive and specific methods for trachoma identification, with potential cost-saving and information benefits. While previous studies have examined the costs of trachoma prevalence surveys, we present the first costing and cost-effectiveness analysis of enhanced trachoma prevalence surveys with ocular swabs and DBS in addition to grading.

**Methodology/Principal findings:**

We calculated the incremental financial cost of enhanced trachoma prevalence surveys with swabs, DBS, and grading using expenditure records from four districts in Tanzania and four districts in Mozambique in 2022. In Tanzania, the cost per cluster of an enhanced survey was $2,337.39 compared to $459.75 for a standard survey. In Mozambique, the cost per cluster of an enhanced survey was $2,147.12, compared to $1,381.46 for a standard survey. We calculated the incremental cost-effectiveness ratio for each method, defined as the ratio of incremental cost to additional instances of trachoma indicators identified, and explored variation in cost-effectiveness via sensitivity analyses. Adding swabs, DBS, or both was cost-increasing and more effective at identification of trachoma indicators than grading alone. In Tanzania, swabs were the most cost-effective method, while DBS was more cost-effective in Mozambique. Swabs and DBS were less cost-effective when combined than individually. The main factor determining cost-effectiveness was sensitivity.

**Conclusions/Significance:**

Adding swabs or DBS to trachoma prevalence surveys can be viable, cost-effective methods for identifying trachoma indicators. The additional costs are commensurate with additional information that would support elimination efforts.

## Author summary

Countries have made progress towards eliminating trachoma, a bacterial blinding disease. To assess trachoma prevalence, countries conduct surveys involving examination for clinical signs of trachoma, which in turn inform the decision to implement mass drug administration of antibiotics. However, clinical examination can result in errors, particularly as the disease becomes rare. Countries have explored alternative methods of identifying trachoma, which include testing swabs of the inner eyelid or dried blood spots (DBS). These methods can be more accurate than clinical examination, but also more costly. We examined the costs of surveys with swabs and DBS in Tanzania and Mozambique compared to surveys with clinical examination alone. We found that adding swabs and DBS to a survey increased costs by 408% in Tanzania and 55% in Mozambique. We also assessed cost-effectiveness, or the extent to which additional costs corresponded to greater trachoma indicator identification, and found that swabs were most cost-effective in Tanzania, while DBS were most cost-effective in Mozambique. Using both swabs and DBS together in a survey added the most costs, with similar gain in trachoma identification compared to using one or the other. Our results will help countries plan for future surveys.

## Introduction

Trachoma, caused by the bacterium *Chlamydia trachomatis (Ct)*, is the leading infectious cause of blindness in the world [1,2]. The World Health Organization (WHO) aims to eliminate trachoma as a global health problem by 2030; as of October 21, 2024, 21 countries have accomplished this goal [1,3]. Over the past two decades, global health programs have made substantial progress in preventing and treating the disease through the WHO-recommended “SAFE” strategy involving **S**urgery for trachomatous trichiasis (TT), **A**ntibiotic administration, **F**acial cleanliness, and **E**nvironmental improvement [1]. As a result of these efforts, the number of people at risk for trachoma-related blindness has decreased by 91.6% [2]. However, trachoma remains a public health problem in 38 countries [1,3] resulting in irreversible blindness or visual impairment of approximately 1.9 million people, with 103 million people living in trachoma-endemic areas still at risk of blindness [1]. Trachoma remains most prevalent among the poorest populations in low-income countries, particularly in sub-Saharan Africa [1,4].

Trachoma has two stages of disease progression. The first stage involves repeated infection with conjunctival strains of *Ct*, which causes trachomatous inflammation—follicular (TF), or active trachoma. TF is most common among young children, with infections peaking at ages 2–5 then decreasing in frequency and duration with age [1,5]. Infection can be spread through direct or indirect contact with eye and nose discharge of infected individuals, as well as by particular species of flies [1,5]. While the immune system can clear a single episode of infection, repeated *Ct* infections lead to conjunctival scarring and eventually the second stage of disease, TT. If left untreated, TT can cause irreversible vision impairment and blindness, which typically affects individuals later in life [1,5]. In addition to the human health cost, the annual economic cost due to lost productivity from trachoma is estimated at 2.9–5.3 billion U.S. dollars (USD), or 8 billion USD when taking the blinding stage of TT into account [1].

In trachoma-endemic districts, public health organizations conduct population-based trachoma prevalence surveys to measure TT and TF prevalence. For a country to reach elimination status, the WHO outlines several requirements, which include a lower than 0.2% prevalence of TT in individuals 15 years old or older, a lower than 5% prevalence of TF in children aged 1–9 which is sustained for at least two years after antibiotic mass drug administration (MDA), as well as a system in place able to identify and manage incident TT cases [1]. If TF prevalence is above the 5% threshold, districts conduct MDA with the antibiotic azithromycin. After MDA, districts continue to monitor trachoma prevalence through surveys such as trachoma impact surveys (TIS), which assess whether additional rounds of MDA are needed according to the 5% TF threshold [6]. Trachoma prevalence surveys like TIS are critical to monitor TF prevalence in trachoma-endemic districts, guide decision-making for implementation of MDA, and eventually achieve elimination.

Currently, WHO guidelines for trachoma prevalence surveys dictate that TF cases be identified by clinical examination using the simplified trachoma grading system, which provides the estimate of TF prevalence on which the MDA implementation decision threshold is based [7]. This process involves a trained grader flipping and examining a child’s conjunctiva for clinical signs of active trachoma, indicated by the presence of five or more lymphoid follicles at least 0.5 mm in diameter [7,8]. However, there are concerns surrounding grading’s accuracy in reflecting the true burden of trachoma, since infections can be asymptomatic, inflammation often appears after a 5–10 day incubation period, and follicles can persist weeks after infection is cleared [5]. Furthermore, grading is subjective and vulnerable to human error, with even experienced graders sometimes disagreeing on presence or absence of TF [9,10]. The correlation between TF and ocular *Ct* infection weakens in low-prevalence and post-MDA settings, due to persistence of clinical signs following treatment and increased difficulties training graders as cases become rare [8,11,12]. Since follicles are not unique to *Ct* infection and may persist weeks after infection clears, TF may overestimate trachoma prevalence, potentially resulting in unnecessary rounds of MDA [8,13,14]. In the literature, grading has had wide variation in sensitivity, specificity, and agreement with other indicators of infection; mean sensitivity of grading for TF in previous studies ranges from 50% to 87%, while mean specificity ranges from 37% to >90% [10,15–18]. Given the wide variability in the reliability of TF as an indicator of ocular *Ct* infection and its reduced accuracy in post-treatment settings, more sensitive and specific assessments of the trachoma burden in these countries are required to support trachoma surveillance and decision-making as more countries near elimination.

In response to the challenges and limitations of relying on clinical signs of TF as an accurate reflection of the *Ct* burden in a population, health ministries, control programs, and other groups have explored the use of alternate diagnostic approaches for assessing trachoma prevalence during surveys. Considered the “gold standard” for detecting *Ct* infection, nucleic acid amplification tests, a polymerase chain reaction (PCR) based assay, have been used to assess ocular swab samples [8,13]. PCR can detect preclinical and asymptomatic infections that do not present as TF, and can verify if infection has cleared when clinical signs persist [19]. However, PCR may miss infections with low bacterial loads [5]. PCR is highly specific, with near 100% specificity and positive predictive values in the literature, while sensitivity is more variable, with estimates ranging from 61% to 100% [8,12,14,17,18]. While costs have limited its widespread use for control programs, with more specific results, PCR testing can avoid unnecessary MDA and diminish overtreatment, in turn incurring long-term cost savings and public health benefits [13,18].

Serological analysis of dried blood spots (DBS) has been used to detect antibodies to *Ct* antigens to assess the level of trachoma transmission, providing an estimate of the force of infection in a community by characterizing seroprevalence by age [11,20–22]. Previous studies have shown that serological data could be useful for post-MDA surveillance [11,21,23]. Antibody seroprevalence increases with age, likely reflecting cumulative *Ct* exposure over time; after MDA, antibody responses decline [24]. As such, including serological testing in a survey can provide insights into historical transmission dynamics, such as if continued exposure is occurring. For example, the absence of antibodies in young children could indicate interrupted transmission [22]. Serology can also detect low-level or subclinical infections that clinical examination might miss. In the literature, serological tests have been sensitive and specific, with >90% sensitivity and specificity ranging from 69% to 98% [11,22,25]. Compared to PCR, serological tests are likely to present a lower cost burden [24]. In trachoma-endemic and post-MDA settings, both PCR and serological tests have produced more sensitive and specific results compared to clinical grading [8,10,11,14,17]. Thus, adding ocular swab and DBS collection to a standard trachoma prevalence survey using clinical examination has the potential to improve efforts towards trachoma elimination [11]. There do not yet exist WHO guidelines surrounding the use of PCR or serology during trachoma prevalence surveys, and more data is needed to determine decision-making thresholds and operationalize these methods for programmatic use [14,23]. Furthermore, adding diagnostic tests to trachoma prevalence surveys could present a substantial cost burden, the extent of which remains largely unknown.

There are a limited number of studies that address the costs of assessing trachoma prevalence, and fewer that evaluate cost-effectiveness. Chen et al. (2011) calculated the cost per district, per cluster, and per person of trachoma prevalence surveys conducted in 165 districts across eight national trachoma control programs in Africa from 2006 to 2010 [26]. Trotignon et al. (2017) assessed the mean survey cost per district, per evaluation unit, per cluster, and per person of baseline trachoma mapping surveys for the Global Trachoma Mapping Project (2012–2016) in 17 countries [27]. Slaven et al. (2020) analyzed the mean total cost per cluster of 8 rounds of TIS and trachoma surveillance surveys (TSS) executed in 187 districts in Amhara, Ethiopia, from 2012 to 2016 [28]. Stelmach et al. (2019) examined the median cost per evaluation unit of TIS/TSS in 11 countries between 2011 and 2018, and TT-only surveys in four countries between 2017 and 2018 [29]. Regarding the costs of testing for *Ct* infection, Harding-Esch et al. (2015) evaluated the mean cost of ocular swab testing against the cost of MDA per census enumeration area in the Gambia [13].

To assist control programs in selecting strategies to identify populations in need of trachoma interventions based on economic rationale, we examine the costs and cost-effectiveness of adding ocular swabbing and PCR testing and DBS collection and testing to trachoma prevalence surveys in addition to standard clinical examination in Tanzania and Mozambique, comparing the additional cost of these methods with the additional information provided regarding the population-level trachoma burden. Furthermore, we evaluate the main drivers of cost differences between the additional biomarkers and how costs might change should measuring these biomarkers become standard practice during trachoma prevalence surveys. To make results more widely applicable, we also explore variation in parameters influencing the cost-effectiveness of each method. As the first comparative costing and cost-effectiveness analysis of trachoma prevalence surveys with *Ct* infection and serological testing, the results from this study will inform national trachoma programs of the financial resources required to utilize these biomarkers for monitoring trachoma as countries reach elimination.

## Methods

### Setting

**Fig 1:**
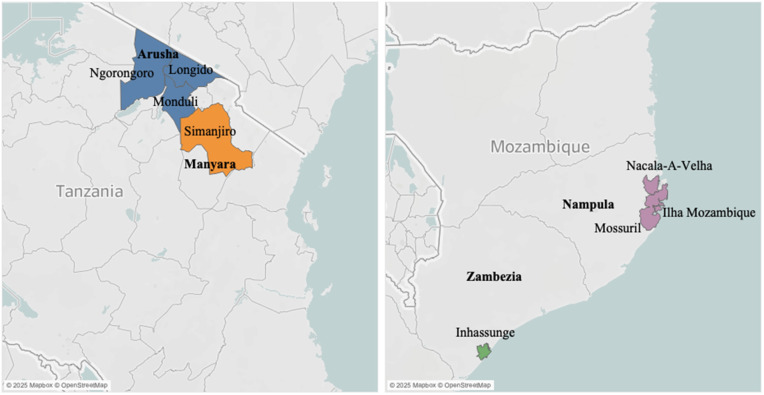
Map of survey region. This map shows the districts included in the surveys mentioned in this study. Colors correspond to region. Shapefiles accessed from https://data.humdata.org/dataset/cod-ab-tza (Tanzania) and https://data.humdata.org/dataset/cod-ab-moz (Mozambique). Contains information from OpenStreetMap and OpenStreetMap Foundation, which is made available under the Open Database License.

This study evaluates the relative cost-effectiveness of a pilot trachoma monitoring program implemented in 2022 that involved both biomarker testing methods (PCR and DBS), hence referred to as enhanced trachoma prevalence surveys, in addition to standard clinical examinations, or standard trachoma prevalence surveys. The implementation setting of enhanced trachoma prevalence surveys was four districts in Nampula and Zambezia regions in Mozambique (Mossuril, Ilha Mozambique, Nacala-A-Velha, and Inhassunge) and four districts in Arusha and Manyara regions in Tanzania (Longido, Monduli, Ngorongoro, and Simanjiro) (Fig 1). These districts in Mozambique and Tanzania reflect trachoma-endemic settings in which MDA has been implemented to reduce TF prevalence, which aligns with other PCR and serological testing scenarios found in the literature [8,11,14].

In Mozambique, 66 districts are endemic for trachoma as of 2019 [30]. A study covering 137 Mozambican districts between July 2012 and May 2015 found that TF prevalence in children aged 1–9 was ≥ 10% in 20 districts and 5.0–9.9% in 28 districts and the prevalence of trichiasis in individuals aged 15 years and above was ≥ 0.2% in 34 districts [31]. Since then, Mozambique has made substantial progress in reducing rates of TF and TT through SAFE efforts, with 14 districts above 5% TF prevalence as of 2022 [4,30,32]. The districts included in this study have displayed persistent and recrudescent TF, with Nacala-A-Velha, Mossuril, and Ilha Mozambique at ≥5% and Inhassunge at >10% TF prevalence prior to the 2022 surveys [4].

In Tanzania, 71 districts were identified as endemic for trachoma during baseline surveillance in 2004–6 [33]. The 2004–6 baseline survey found a 25.4% prevalence of TF and 2.7% prevalence of TT, with an estimated 12.5 million Tanzanians at risk for trachoma [13]. Due to SAFE interventions, Tanzania has greatly reduced prevalence, with 10 districts remaining above the 5% threshold as of 2022 [4]. Overall, Tanzania has been successful in implementing the SAFE strategy and reaching elimination status in most districts. However, the continued persistence of TF in certain districts, including those in this study, highlights the need for more targeted interventions [34]. Prior to the 2022 surveys, Monduli, Ngorongoro, and Simanjiro had ≥ 5% and Longido had > 10% TF prevalence [4].

### Enhanced trachoma prevalence surveys

The implementation of enhanced trachoma prevalence surveys and sample processing were distinct in the two country settings. In Mozambique, the enhanced trachoma prevalence surveys covered 24 clusters per evaluation unit (EU), which involved ocular swab and DBS sample collection. An EU is the level at which survey results are typically reported, corresponding to the population being surveyed and representing a population unit of 100,000–250,000 [35]. Since the number of clusters per EU fell within the WHO guidelines of 20–30 for a TIS, the Mozambique surveys were considered to be “enhanced TIS” [36]. For full details on the enhanced TIS in Mozambique, please see Sitoe et al. (2024) [37]. Briefly, during the enhanced TIS, 4,841 children aged 1–9 were examined for clinical signs of trachoma using the simplified grading system, in which trained graders examine the upper tarsal conjunctiva for the presence of five or more follicles indicating TF [7]. All individuals with identified TF were offered 1% tetracycline eye ointment to apply twice daily for 6 weeks. Two conjunctival swab samples were also collected from each child, one dry and one using preservation media. Swab samples were then shipped to the Proctor Foundation at University of California, San Francisco (UCSF) and the dry samples were processed using the iAMP *Ct* Detection Kit (Atila, CA, United States), an isothermal PCR assay intended for the qualitative detection of *Ct* infection [8]. DBS were collected from each child via a finger prick procedure and then shipped to the United States Centers for Disease Control and Prevention (CDC) and evaluated for antibodies against *Ct* antigens Pgp3 and Ct694 using a multiplex bead assay [22].

In Tanzania, enhanced trachoma prevalence surveys were implemented as part of an ongoing sentinel site monitoring activity, covering 10 clusters per EU, which is fewer than is covered during a TIS [36]. During the enhanced surveys, a single conjunctival swab and DBS were collected from around 2,022 children aged 1–9 following trachoma grading. As in Mozambique, individuals with identified TF were offered tetracycline eye ointment. All samples were processed at Muhimbili National Hospital in Dar es Salaam using the Cepheid GeneXpert CT/NG (Cepheid, Sunnyvale, CA), a rapid real-time PCR nucleic acid amplification test for ocular swabs and a newly developed lateral flow assay to detect antibodies to *Ct* Pgp3 antigen in DBS [38]. This was relevant for costing purposes as multiplex bead assay is more expensive and resource-intensive than lateral flow assay [38,39]. DBS and swab sampling kits were donated in both countries by the CDC.

### Ethics

This secondary economic evaluation of a health intervention did not involve health data with any identifying information; data consisted solely of cost information and aggregate estimates of sensitivity, specificity, and acceptability. As such, this study was not considered to be human subjects research. Regarding the surveys mentioned in this study, approvals were obtained from the Mozambique National Bioethics Committee for Health (462/CNBS/22), the UCSF Institutional Review Board (21–35587), and the London School of Hygiene & Tropical Medicine (16105) for Tropical Data support [37]. Parents/carers of all children screened provided informed consent, and children aged 6–9 years provided assent. For each child, informed consent was recorded both on household level consent forms signed by parents/carers and electronically on the Tropical Data App. Like the economic researchers, CDC staff did not have access to identifying information. The economic evaluation was conducted in collaboration with health ministries, who gave permission to publish results.

### Cost data collection and analysis

The purpose of this study was to evaluate the incremental financial cost to the implementer of enhanced trachoma prevalence surveys in Tanzania and Mozambique, as well as the relative cost-effectiveness of the alternative trachoma evaluation methods in terms of additional information provided relevant to MDA decision-making. In our economic evaluation of this health intervention, we adhered to the 2022 edition of the consolidated health economic evaluation reporting standards [40]. The economic researchers worked in close collaboration with RTI International (RTI) epidemiologic researchers and the United States Agency for International Development (USAID) Act to End NTDs | East program, led by RTI, which supported the health ministries in implementing enhanced trachoma prevalence surveys in Tanzania and Mozambique. We obtained retrospective cost data from financial expenditure records produced by RTI financial analysts in QuickBooks [41]. We also collected expenditure records from CDC, which contributed supplies for surveys in both countries and conducted the DBS testing for Mozambique, and UCSF, which likewise contributed supplies for enhanced TIS and conducted swab testing for Mozambique. The Act to End NTDs | East program also provided data on the number of people who were screened in each district. Data on laboratory costs were provided by CDC, UCSF, and Muhimbili National Hospital. All cost data were provided immediately following the completion of fieldwork.

To compare the costs of enhanced trachoma prevalence surveys with those of standard surveys, we also obtained cost data for standard TIS in each country. For Mozambique, all costs for the enhanced TIS in the four EUs were obtained, and then actuals associated with a standard TIS were extracted for comparison from the three contiguous EUs in Nampula (Ilha Mozambique, Mossuril, Nacala-A-Velha). For Tanzania, however, the standard and enhanced survey data were distinct. We used data from a standard TIS conducted in Ngorongoro from February to March 2022 for comparison because there was no standard TIS conducted in Tanzania that overlapped with the enhanced survey activity. Actual expenditure data were used wherever possible; however, in the case of Tanzanian standard TIS data, budgets were used due to data availability constraints. Since TIS is regularly conducted in Tanzania with predictable costs, we do not anticipate this factor to affect our results significantly. Standard TIS data is only reflected in aggregate estimates of total survey costs for comparison purposes; all data presented in the following cost and cost-effectiveness analyses of different trachoma evaluation methods was conducted using enhanced survey expenditure data.

While in practice, the enhanced trachoma prevalence surveys in our data involved both ocular swabs and DBS, we evaluated four scenarios to isolate the costs and indicators associated with each trachoma evaluation method: (i) clinical grading alone, (ii) clinical grading with ocular swabbing, (iii) clinical grading with DBS, and (iv) clinical grading with both ocular swabbing and DBS. Clinical grading alone is the current standard for prevalence surveys and thus represents the baseline for costs budgeted for a country to assess trachoma prevalence. To calculate the cost of each evaluation method, we applied an ingredients costing approach to expenditure records [42,43], which involved summing the cost of each line item to get the total cost of the survey. We assigned each line item a category based on the phase of activity and the type of line item to which it pertained (Table 1), and grouped line items by evaluation method: grading, ocular swab, or DBS, with some items applicable to multiple methods. For the full cost dataset, please see S1 Data. Following precedent in the literature, we excluded administrative costs from implementing partners as well as costs that would have occurred in the absence of an enhanced trachoma prevalence survey, such as the base salaries of staff [27,29]. The costs of donated supplies and equipment were included to account for the full cost of conducting the survey if such items were not donated in the future. Lastly, our analysis focused exclusively on the financial cost of the survey; economic costs were not considered.

**Table 1:** Cost data categories and examples of line items by phase of activity and type of item. This table presents how line items were categorized within the cost data and provides examples of which items corresponded to each category. Each line item was assigned a single “activity” and “type” according to these categories for the costing analysis.

	Category name	Examples of line items
Phase of activity	Preparation	Pre-visit, site sensitization, survey planning
	Training	Training of local survey teams and supervisors, includes cost of swab and DBS consultant.
	Survey implementation	Fieldwork and sample collection
	Sample processing	Laboratory costs for swab and DBS testing
Type of line item	Communication	Radio airtime
	Per diem	Per diems for trainers, graders, technicians, etc., including meals and lodging costs.
	Supplies and equipment	Eye swab, tube, contact activated lancet, etc., including shipping costs.
	Travel	Vehicle rental, fuel, in-country air tickets, taxi service

Our outcome of interest for the costing analysis is the difference in financial cost of an enhanced trachoma prevalence survey compared to a standard survey within a survey cluster for each country, considered from the perspective of the survey implementer. We selected the cluster level because it was the most proportionate unit of measurement between the Tanzania and Mozambique settings for this study, as there were 10 clusters per EU surveyed in Tanzania and 24 clusters per EU surveyed in Mozambique. We also report cost per EU to facilitate comparison of our results with others in the literature, noting that the sample sizes were different in each country. To account for different sample sizes, we also calculated the cost per person screened within an EU, which is the cost per EU divided by the sample size within the EU. Lastly, to account for U.S.-based sample processing for Mozambique, we also conducted a hypothetical scenario analysis to consider how costs would change if sample processing were to occur locally by substituting costs related to sample shipping and processing at UCSF and CDC with estimated local laboratory costs.

We collected costs in the currency in which they were spent at the time of expenditure. If expenditures were in local currency, we converted costs to USD using the average exchange rate over the duration of the survey reported by XE.com [44]. Additionally, for most line items that were procured in-country, an 18% value added tax was applied. Since all cost data spanned a single year (2022), a discount rate was not applicable. We report all results in 2022 USD.

### Cost-effectiveness analysis

To evaluate cost-effectiveness of each enhanced trachoma evaluation method, defined in this study as the extent to which the additional costs are commensurate with the additional information provided regarding the trachoma burden within a population, we calculated the incremental cost-effectiveness ratio (ICER) [45]. The ICER, calculated as in Equation 1, summarizes the relationship between the incremental cost of the enhanced method and the additional instances of trachoma indicators identified. Importantly, effectiveness is defined in terms of trachoma indicators to acknowledge that the results provided by clinical grading (TF), testing for current ocular *Ct* infection, and seroprevalence identified through serological testing reflect different stages of the natural history of trachoma and thus serve as different indicators of the trachoma burden in a population. Despite this, we selected the ICER to be a useful estimate to assess cost-effectiveness, aligning with similar cost-effectiveness analyses in the literature such as Sekandi et al. (2015), Odongo et al. (2023), and Zelman et al. (2018) [46–48]. In this study, cost-effectiveness is evaluated based on the extent to which additional costs correspond to additional information that could be useful for control programs. We further reflect on this choice in the discussion.

#### Equation 1. Incremental cost-effectiveness ratio (ICER).


$$ICER=\frac{C_{1}-C_{0}}{T_{1}-T_{0}}$$ (1)

In Equation 1, $\begin{array}{c} \;C_{1} \end{array}$ is the cost per person of the enhanced method (swab, DBS, or both swab and DBS combined), $\begin{array}{c} \;C_{0} \end{array}$ is the cost per person of clinical grading, $T_{1}$ is the instances of trachoma indicators identified using the enhanced method (swabs, DBS, or both swabs and DBS), and $\;T_{0}$ is the instances of trachoma indicators identified using clinical grading.

C is calculated as:

#### Equation 2. Cost per person of a trachoma evaluation method.


$$\begin{array}{c} C=A\;\left\{C_{test}+C_{trt}\left[(Pr\;*Se)+\;\left(1-Pr)(1-Sp)\right)\right]\right\} \end{array}$$ (2)

In Equation 2, A is the percentage of the population accepting the evaluation method, $C_{test}$ is the unit cost of the enhanced method, $C_{trt}$ is the unit cost of antibiotic treatment for trachoma, Pr is the prevalence of trachoma in the population, Se is the sensitivity of the evaluation method, and Sp is the specificity of the evaluation method.

Lastly, T is calculated as:

#### Equation 3. Number of instances of trachoma indicators identified using a trachoma evaluation method.


T=A*Pr*Se (3)

Due to the distinct implementation contexts of enhanced surveys in Tanzania and Mozambique, we conducted separate analyses for each country. While in practice, enhanced surveys utilized both ocular swab and DBS testing for identifying trachoma, we also calculated ICER values for testing scenarios of ocular swabs without DBS, and DBS testing without ocular swabs, to evaluate the relative cost-effectiveness of each biomarker. Wherever possible, we used results from the enhanced surveys as inputs in the ICER equation, which provided values for A, Se, and Sp. Results from our costing analysis for average cost per person surveyed were used as input values for $C_{test}$. For parameters Pr and $C_{trt}$, we used values from the literature that most closely aligned with the country context of the surveys [31,49,50]. For the actual parameter values used in the cost-effectiveness analysis, please see Input Data File 1 (https://doi.org/10.5281/zenodo.14511677).

### Sensitivity analysis

In calculating costs and cost-effectiveness of the enhanced methods, we adopted several assumptions that created a range of uncertainty surrounding our estimates. Additionally, the novel design of enhanced survey activities and the context-driven nature of survey implementation meant there existed a level of uncertainty inherent in the parameter values. To explore the drivers of uncertainty in estimates for cost per person tested ($C_{test}$) and ICER values, we conducted deterministic and probabilistic sensitivity analyses on the components of the ICER equation (Equation 1) for each country [51,52]. The parameter values specified in the cost-effectiveness analysis served as the point estimates for the sensitivity analyses. For each evaluation method (swabs, DBS, swabs and DBS combined), we varied each component of the ICER equation (A, $C_{test}$, $C_{trt}$, Pr, Se, Sp) as well as the category components of $C_{test}$, where $C_{test}$ is the sum of the types of costs described in Table 1 divided by the total survey sample size. We defined the range of possible values for each parameter to be the minimum and maximum values found in the fieldwork data and country-specific literature [10,31,49,50].

We first ran deterministic sensitivity analysis to determine which parameters were driving variation in ICER values. We varied each parameter according to its minimum and maximum for each method (grading, swabs, DBS, swabs and DBS combined) holding all other parameters constant, which resulted in a unique ICER for each method-parameter pair. Using the same range of parameter values and assuming a PERT distribution for each parameter, we generated a Latin hypercube sample [53] of 10,000 rows and conducted a probabilistic sensitivity analysis with 10,000 runs. The results show the full range of plausible ICER outcomes for each enhanced method. We report the full range of results and 95% confidence intervals using 2.5^th^ and 97.5^th^ centiles. Lastly, we ran quantile regression on the probabilistic sensitivity analysis results to assess the predictive effect of influential parameters on the ICER, estimating 0.1, 0.5, and 0.9 quantile weights.

We performed all data analysis and visualization involved in the costing, cost-effectiveness, and sensitivity analysis components of this study using R version 4.5.0 [54]. Please see Code File 1 and Code File 2 (https://doi.org/10.5281/zenodo.14511677) for the R code used to perform the sensitivity analyses.

## Results

### Costs of enhanced trachoma prevalence surveys

In Tanzania, the cost per cluster for enhanced trachoma prevalence surveys with clinical grading, swabs, and DBS was $2,337.39, or $23,373.89 per EU, compared to the budget of $459.75 per cluster, or $13,792.50 per EU, for a standard survey with grading only. Per cluster costs for the Tanzania enhanced survey were 408.4% higher than a standard survey with clinical grading, while per EU costs were a 69.5% increase from the baseline. In Mozambique, the cost per cluster for enhanced TIS was $2,147.12, or $51,530.54 per EU, while the mean cost of a standard TIS with grading only was $1,381.46 per cluster, or $33,155.13 per EU. Compared to a standard TIS in Mozambique, per cluster and per EU costs were 55.4% higher for enhanced TIS.

Table 2 shows the distribution of costs according to the categories outlined in Table 1. By cost type, supplies and equipment ($1,197.23 per cluster) and per diems ($669.72 per cluster) accounted for the largest share of costs. In Mozambique, the greatest cost type was per diems ($999.06 per cluster), followed by supplies and equipment ($586.93 per cluster) and travel costs ($561.12 per cluster). In both countries, communication costs were minimal, with no communication costs incurred at all in Mozambique. By phase of activity, training ($1,003.52 per cluster) and sample processing ($931.99 per cluster) made up the greatest proportion of costs in Tanzania (Table 2). In Mozambique, survey implementation was the most expensive phase at $1,415.16 per cluster, followed by sample processing ($468.76 per cluster).

**Table 2:** Enhanced trachoma prevalence survey costs by phase of activity and type of item in Tanzania and Mozambique. This table shows the breakdown of total costs in each country by the categories described in Table 1: phase of activity and type of item. Percentages were calculated as the proportion of expenditures corresponding to each phase and type.

Tanzania					
Phase/Type	Communication	Per diem	Supplies/equipment	Travel	Total
Preparation	0.0%	1.2%	0.1%	0.0%	1.4%
Sample processing	0.0%	0.0%	39.9%	0.0%	39.9%
Survey implementation	0.5%	4.3%	10.8%	0.2%	15.8%
Training	0.0%	23.1%	0.4%	19.4%	42.9%
Total	0.5%	28.7%	51.2%	19.6%	100.0%
**Mozambique**					
Phase/Type	Communication	Per diem	Supplies/equipment	Travel	Total
Preparation	0.0%	0.0%	5.1%	0.8%	5.9%
Sample processing	0.0%	10.8%	11.1%	0.0%	21.8%
Survey implementation	0.0%	30.7%	10.4%	24.8%	65.9%
Training	0.0%	5.1%	0.7%	0.5%	6.3%
Total	0.0%	46.5%	27.3%	26.1%	100.0%

Within the enhanced survey cost data, we isolated line items that pertained to each method to determine the costs of grading only, grading and swabs, grading and DBS, or grading and both swabs and DBS (Table 3). The cost per EU for each evaluation method was greater in Mozambique than in Tanzania, while cost per cluster and cost per person was higher in Tanzania where fewer clusters were surveyed. In both countries, the greatest costs were for the combined swab and DBS method, which increased per cluster costs by 88.9% from clinical grading alone in Tanzania and by 90.2% in Mozambique. Swabs were more expensive than DBS in both countries; swabs increased total survey costs by 49.0% in Tanzania and 60.8% in Mozambique, while DBS increased survey costs by 45.8% in Tanzania and by 32.8% in Mozambique. It is important to note that the “grading only” estimates were produced by isolating clinical grading costs within the enhanced trachoma prevalence survey data. As such, for Tanzania, “grading only” reflects clinical grading costs incurred during the enhanced survey activity, distinct from the standard TIS data. For Mozambique, since the standard TIS was part of the enhanced TIS, grader-only costs reflect standard TIS costs with the exception of one additional district (Inhassunge) included in the enhanced TIS data.

**Table 3:** Cost per EU, cost per cluster, and cost per person surveyed using each trachoma evaluation method in Tanzania and Mozambique (2022 USD). This table shows cost per EU, cost per cluster, and cost per person of each trachoma evaluation method for each country. While in practice, surveys involved all three evaluation methods, costs pertaining to each evaluation method (grading, swabs, or DBS) were isolated as described in methods. Some costs overlapped between methods. Table values were calculated by dividing total costs by number of EUs, clusters, and individuals screened in the surveys.

Evaluation method	Tanzania			Mozambique		
Cost per EU	Cost per cluster	Cost per person	Cost per EU	Cost per cluster	Cost per person
Grading, swabs, and DBS	$23,373.89	$2,337.39	$46.24	$51,530.54	$2,147.12	$42.58
Grading and swabs	$18,442.53	$1,844.25	$36.48	$43,565.06	$1,815.21	$36.00
Grading and DBS	$18,048.27	$1,804.83	$35.70	$35,977.60	$1,499.07	$29.72
Grading only	$12,376.94	$1,237.69	$24.48	$27,098.45	$1,129.10	$22.39

Since swab and DBS sample processing differed by country, occurring at a local laboratory in Tanzania and overseas at UCSF and CDC for Mozambique, we conducted a hypothetical scenario analysis for Mozambique to consider how costs might change if sample processing were performed locally. For this scenario, we substituted costs related to shipping and processing of samples at UCSF and CDC with the costs of sample processing in Tanzania. Since lateral flow assay was used to process DBS samples in Tanzania, the hypothetical scenario analysis also involves lateral flow assay in place of the more expensive multiplex bead assay used for testing samples from Mozambique. Accordingly, the Mozambique local laboratory scenario would result in lower costs for all diagnostic methods except for grading (Table 4). By conducting laboratory testing locally, we estimate that costs would decrease by 15.2% for swabs, 1.6% for DBS, and 6.9% for both swabs and DBS compared to the realized costs in Mozambique. This scenario also reduced the cost difference between the two methods, with swabs now 4.3% more expensive than DBS. In the local scenario, swabs would result in a cost increase of 36.4% from grading alone, compared to 60.8% with UCSF and CDC testing, while DBS would result in a cost increase of 30.7% instead of 32.8%.

**Table 4:** Cost per EU, cost per cluster, and cost per person surveyed using each trachoma evaluation method during enhanced TIS in Mozambique (2022 USD), comparing the actual costs incurred to a hypothetical local laboratory sample processing scenario. This table shows the cost per EU, cost per cluster, and cost per person for each trachoma evaluation method for the realized Mozambique costs and the hypothetical scenario analysis substituting local laboratory costs. Mozambique actual costs reflect the same estimates for Mozambique in Table 3.

Evaluation method	Mozambique Actual Costs			Mozambique Local Laboratory Scenario Analysis		
	Cost per EU	Cost per cluster	Cost per person	Cost per EU	Cost per cluster	Cost per person
Grading, swabs, and DBS	$51,530.54	$2,147.12	$42.58	$47,971.84	$1,998.83	$39.64
Grading and swabs	$43,565.06	$1,815.21	$36.00	$36,949.19	$1,539.55	$30.53
Grading and DBS	$35,977.60	$1,499.07	$29.72	$35,414.78	$1,475.62	$29.26
Grading only	$27,098.45	$1,129.10	$22.39	$27,098.45	$1,129.10	$22.39

### Cost-effectiveness of enhanced trachoma evaluation methods

To assess the cost-effectiveness of additional biomarkers implemented during enhanced trachoma prevalence surveys compared to standard surveys, we calculated the ICER of each method for both countries using Equation 1. Table 5 shows the incremental cost per person screened, incremental effect, and ICER for each evaluation method using point estimate parameter values, which were selected from the costing results, fieldwork results, and the literature [2,35,49].

**Table 5:** Incremental cost-effectiveness ratio (ICER) point estimates for swabs and DBS as part of enhanced trachoma prevalence surveys in Tanzania and Mozambique, compared with grading-only costs (2022 USD). This table shows point estimates for incremental cost, additional instances of trachoma indicators identified, and incremental cost-effectiveness ratios for the three evaluation methods in Tanzania and Mozambique. Values were calculated according to Equations 1–3 described in methods.

Evaluation method	Tanzania			Mozambique		
	Incremental cost per person surveyed	Additional instances of trachoma indicators identified	ICER	Incremental cost per person surveyed	Additional instances of trachoma indicators identified	ICER
Swabs	$10.95	0.023	475.25	$9.41	0.016	583.62
DBS	$10.17	0.018	572.58	$3.73	0.012	323.05
Swabs and DBS	$24.15	0.023	1048.29	$15.17	0.016	949.34

The positive ICER values for all three evaluation methods show that the addition of swabs, DBS, or both to a trachoma prevalence survey increased survey costs and provided additional trachoma indicator identification compared to a standard grading-only survey. For both Tanzania and Mozambique, the higher ICERs for the combined swab and DBS method indicate that swabs and DBS individually provided a more balanced ratio of incremental costs to additional information than when used together during enhanced surveys. The combined swab and DBS method resulted in the greatest incremental costs, with similar indicator identification as the individual methods. In Tanzania, swabs were the most cost-effective evaluation method, while DBS was more cost-effective than swabs in Mozambique. As previously shown, local sample processing in Mozambique could reduce the cost of swabs, so we evaluated the cost-effectiveness outcome under this scenario as well. In the theorized local laboratory scenario, the incremental cost per person of swabs was $4.52, which is 52% lower than with international sample processing. The resulting ICERs for swabs and DBS would be $280.5 and $289.5 per instance of trachoma indicators identified, respectively, making the biomarkers closer in cost-effectiveness in the local scenario, similar to the Tanzanian case.

### Sensitivity analyses

**Fig 2:**
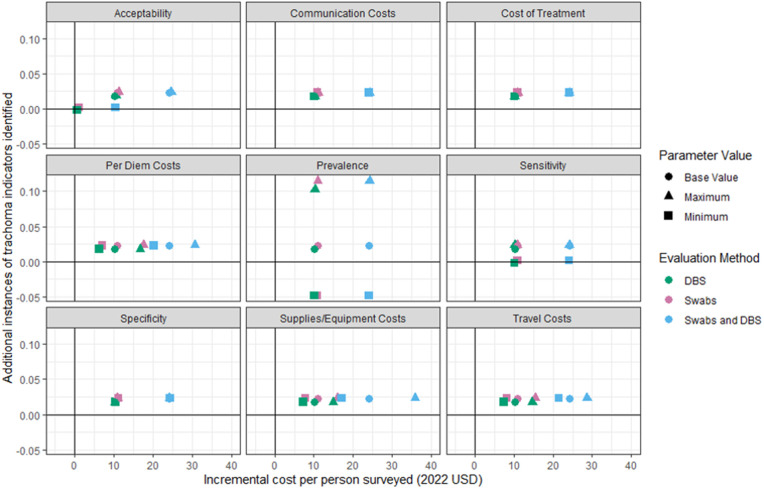
Tanzania deterministic sensitivity analysis results plotted on cost-effectiveness planes, showing the change in incremental cost and incremental effect corresponding to a change in the value of a given parameter, holding all other parameters constant. This figure shows the results from the deterministic sensitivity analysis for Tanzania plotted on individual cost-effectiveness planes for each parameter value. Each plot corresponds to the parameter that was varied according to its maximum and minimum values, holding all other parameters constant. Each point on the plot represents an ICER value, where color corresponds to the evaluation method and shape corresponds to the value used in the ICER calculation.

**Fig 3:**
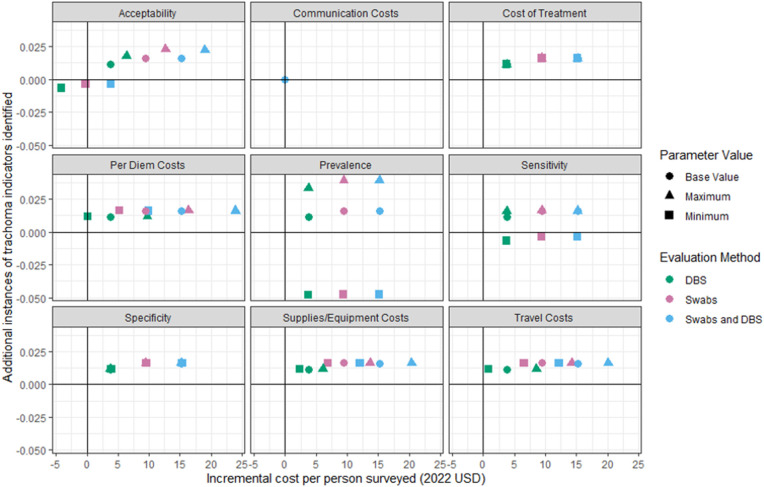
Mozambique deterministic sensitivity analysis results plotted on cost-effectiveness planes, showing the change in incremental cost and incremental effect corresponding to a change in the value of a given parameter, holding all other parameters constant. This figure shows the results from the deterministic sensitivity analysis for Mozambique plotted on individual cost-effectiveness planes for each parameter value. Each plot corresponds to the parameter that was varied according to its maximum and minimum values, holding all other parameters constant. Each point on the plot represents an ICER value, where color corresponds to the evaluation method and shape corresponds to the value used in the ICER calculation.

The ICERs depended greatly on the implementation context of the enhanced trachoma prevalence surveys, with differentiating factors including the smaller number of clusters per EU in Tanzania and U.S.-based sample processing for Mozambique. To account for uncertainty in ICER point estimates, we explored a range of possible cost-effectiveness outcomes through sensitivity analyses. Figs 2 and 3 show the deterministic sensitivity analysis results by ICER parameter on a cost-effectiveness plane. For both Tanzania and Mozambique, the incremental cost and incremental effect were always positive, in the first quadrant of the cost-effectiveness plane, for all parameters when at their base or maximum values. For Tanzania, using minimum values for acceptability or sensitivity resulted in a negligible incremental effect for all three evaluation methods, while prevalence produced a negative incremental effect when at its minimum value. Mozambique exhibited similar results; acceptability, sensitivity, and prevalence each resulted in negative incremental effectiveness when at their minimum values. For Mozambique, per diem and travel costs resulted in a near-zero incremental cost for DBS when at their minimum values. For both countries, when acceptability was at its minimum value, the incremental costs and effects of the evaluation methods were reduced, falling along the x-axis, origin, and sometimes third quadrant of the cost-effectiveness plane. Variation in communication costs, cost of treatment, and specificity resulted in no change in incremental cost or effect.

Using the deterministic sensitivity analysis results, we calculated the difference between the new and baseline ICER values and extent of spread to determine the proportion of variation in ICER values explained by each parameter (Table 6). Overall, sensitivity was the dominant driver of uncertainty in ICER values across evaluation methods in both countries, except for DBS in Mozambique. For Mozambique DBS, per diem costs accounted for the greatest proportion of variation, followed by sensitivity and travel costs. Apart from the outlier of Mozambique DBS, sensitivity accounted for most of the variation in ICER values, with the remaining parameters having a negligible effect.

**Table 6:** Percentage of variation in the incremental cost-effectiveness ratio (ICER) value explained by each parameter, by country and evaluation method. This table shows the percentage of variation in ICER values accounted for by each parameter by evaluation method and country in the deterministic sensitivity analysis results. Columns may not sum to 100% due to rounding.

Parameter	Tanzania			Mozambique		
Swabs	DBS	Swabs and DBS	Swabs	DBS	Swabs and DBS
Sensitivity	98.5%	95.0%	86.3%	88.6%	31.4%	82.8%
Acceptability	0.1%	2.5%	13.0%	2.2%	3.4%	11.5%
Per Diem Costs	0.5%	1.1%	0.1%	4.1%	35.3%	2.5%
Supplies/Equipment Costs	0.3%	0.6%	0.3%	1.5%	5.4%	0.9%
Prevalence	0.3%	0.3%	0.2%	1.6%	1.9%	1.6%
Travel Costs	0.3%	0.5%	0.0%	2.0%	22.7%	0.8%
Specificity	0.0%	0.0%	0.0%	0.0%	0.0%	0.0%
Communication Costs	0.0%	0.0%	0.0%	0.0%	0.0%	0.0%
Cost of Treatment	0.0%	0.0%	0.0%	0.0%	0.0%	0.0%

Table 7 shows the median ICER values and 95% confidence intervals by trachoma evaluation method and country, displaying a wide range of cost-effectiveness outcomes. Among the three evaluation methods, DBS had the greatest variation, particularly in Mozambique, where the lower bound of the 95% uncertainty interval was negative driven by negative incremental effectiveness. Overall, Mozambique had a wider range of ICER outcomes than Tanzania, demonstrating greater uncertainty inherent in the Mozambique setting. In both countries, the combined swab and DBS method had a higher ICER than the individual methods across the full range of results, which indicates lower cost-effectiveness compared to swabs or DBS alone.

**Table 7:** Summary of probabilistic sensitivity analysis of incremental cost-effectiveness ratios (ICER) results by country, evaluation method, and centile (2022 USD). This table shows the ICER point estimates and 95% confidence intervals calculated from the deterministic sensitivity analysis results for each country and evaluation method.

Evaluation method	Tanzania			Mozambique		
	ICER 95% uncertainty interval (lower bound)	Observed ICER value	ICER 95% uncertainty interval (upper bound)	ICER 95% uncertainty interval (lower bound)	Observed ICER value	ICER 95% uncertainty interval (upper bound)
Swabs	152.75	475.25	2,370.20	101.73	583.62	3,238.43
DBS	146.52	572.68	3,813.51	-1,691.96	323.05	3,035.72
Swabs and DBS	478.26	1,048.29	5,331.72	429.21	949.34	5,639.82

**Fig 4:**
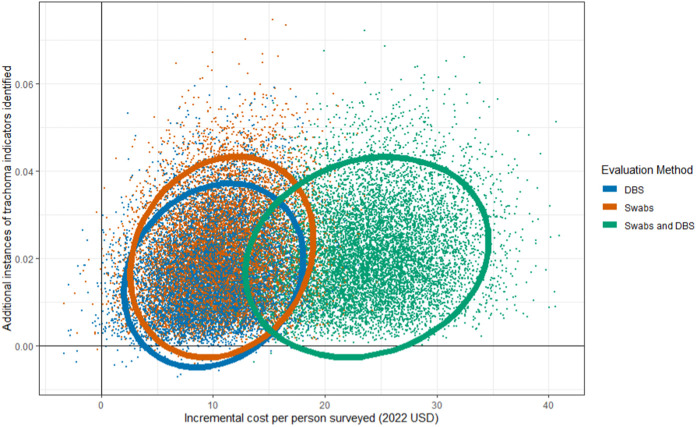
Tanzania probabilistic sensitivity analysis results showing incremental cost per person surveyed and additional instances of trachoma indicators identified for each of the three enhanced evaluation methods plotted on a cost-effectiveness plane. This figure shows all 10,000 runs of the probabilistic sensitivity analysis for Tanzania. Each point represents a single ICER value, with color corresponding to evaluation method. The ellipse encompasses the 95% confidence interval.

**Fig 5:**
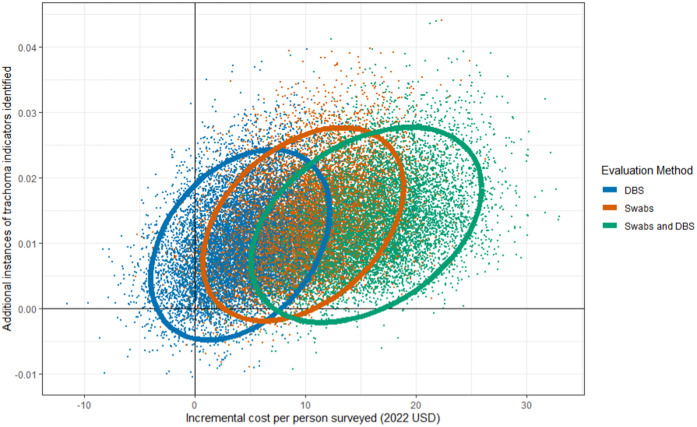
Mozambique probabilistic sensitivity analysis results showing incremental cost per person surveyed and additional instances of trachoma indicators identified for each of the three enhanced evaluation methods plotted on a cost-effectiveness plane. This figure shows all 10,000 runs of the probabilistic sensitivity analysis for Mozambique. Each point represents a single ICER value, with color corresponding to evaluation method. The ellipse encompasses the 95% confidence interval.

In addition to minimum and maximum values, we explored the full range of plausible cost-effectiveness outcomes based on the ICER parameters’ probability distributions. Figs 4 and 5 show the results of the probabilistic sensitivity analysis conducted on ICER parameters for the three evaluation methods on cost-effectiveness planes. For both Tanzania and Mozambique, the combined swab and DBS measure incurred the highest incremental cost with no gain in incremental effectiveness compared to swabs without DBS. Tanzania had considerable overlap in cost-effectiveness outcomes for swabs and DBS, with swabs slightly more cost-effective overall than DBS. In Mozambique, there was greater spread in ICER outcomes between swabs and DBS, as well as a diverse range of cost-effectiveness outcomes for DBS spanning all four quadrants of the cost-effectiveness plane. For Mozambique DBS, 15.2% of ICER results were negative, compared to 1.7% of Mozambique ICER values for swabs and 1.3% for swabs and DBS combined. The greater share of negative ICERs suggests that DBS tends to be more cost-effective than swabs in Mozambique, as most of the difference in trachoma indicator identification is offset by cost savings.

The deterministic sensitivity analysis results highlighted the importance of test sensitivity on variation in the ICER. Given this finding, we conducted quantile regression analysis to assess the predictive effect of test sensitivity on 0.1, 0.5, and 0.9 quantiles of ICER outcomes. Here, a change in sensitivity refers to a change in the difference in sensitivity between the biomarker and clinical grading. For Tanzania, the predictive effect of a one percent increase in sensitivity was a decrease in the absolute value of ICER, or a move toward the origin of the cost-effectiveness plane, by 766.2, 1,695.0, and 7,556.4 for 0.1, 0.5, and 0.9 quantiles respectively, which was statistically significant (p < 0.001) for all quantiles. For Mozambique, we added per diem costs to the model due to its influence on the cost-effectiveness of DBS based on the deterministic sensitivity analysis results. Here, a one percent increase in sensitivity was associated with a decrease in the absolute value of ICER by 629.55, 2,514.9, and 12,581 for 0.1, 0.5, and 0.9 quantiles, and a unit increase in per diem costs was associated with an increase in the absolute value of ICER by 71.68, 104.54, and 204.11 for 0.1, 0.5, and 0.9 quantiles. The coefficients on sensitivity and per diem costs were all statistically significant (p < 0.001). Given their limited influence on cost-effectiveness, we did not include other ICER parameters in the model nor assess their statistical significance.

## Discussion

We found that adding ocular swab and DBS collection and processing to a trachoma prevalence survey was cost-increasing and more effective at identifying the presence of trachoma indicators within a population than a standard trachoma prevalence survey using clinical grading alone. The enhanced survey cost $2,337.39 per cluster in Tanzania and $2,147.12 per cluster in Mozambique, while a standard survey cost $459.75 per cluster in Tanzania and $1,381.46 per cluster in Mozambique. The unique implementation contexts for surveys in each country were important factors influencing cost outcomes, which included Mozambique sample processing occurring in the U.S. instead of locally, and surveys in Tanzania covering 10 clusters per EU instead of 24. While the cost per cluster and cost per person of enhanced surveys were higher in Tanzania, the cost per EU and total cost of survey activities were higher in Mozambique. Our costing analysis of the biomarkers found that ocular swabs were more expensive than DBS, particularly in Mozambique where sample processing was conducted in the U.S. In Tanzania, swabs were the most cost-effective evaluation method, while DBS was more cost-effective in Mozambique. However, under a hypothetical scenario analysis of local laboratory testing in Mozambique, swabs would be more cost-effective. In both countries, the most expensive evaluation method was swabs and DBS combined, with similar corresponding gain in trachoma identification as the individual biomarkers.

The greatest proportion of costs by type were per diems in Mozambique and supplies and equipment in Tanzania. By phase of survey activities, the greatest cost categories were survey implementation in Mozambique and training in Tanzania. While Mozambique sample processing was more expensive than Tanzania overall, this activity made up a smaller proportion of total costs due to higher survey implementation costs, which can be mainly attributed to higher per diems in the Mozambique setting. Survey implementation in Mozambique incurred a 282.2% higher cost per cluster than in Tanzania, with per diems costing 49.2% more per cluster in Mozambique than Tanzania. As such, when considering the hypothetical scenario of local laboratory testing in Mozambique, which we estimated would lower total survey costs by 6.9%, actual local laboratory costs may be higher in practice. Additionally, since multiplex bead assay is more expensive than lateral flow assay, we would expect DBS processing costs to be higher than estimated here if multiplex bead assay were used [38,39].

Our data showed that enhanced trachoma prevalence surveys in Tanzania and Mozambique involved a substantial cost increase from the baseline of a standard survey with clinical grading. The costs of standard trachoma prevalence surveys in the standard TIS data used for comparison were also above or within the upper range of previous costs reported in the literature, suggesting that costs of a standard survey may have increased as well [26,27,29]. The cost per cluster of standard TIS in Tanzania as budgeted was $460 per cluster, or $13,792.50 per EU, which is higher than the inflation-adjusted median of $10,080 per EU reported by Stelmach et al. (2019) and the inflation-adjusted value of $360 per cluster, or $9,372 per EU, reported for Tanzania by Trotignon et al. (2017) [27,29]. In Mozambique, the cost per EU for a standard TIS was $33,155 per EU ($1,381 per cluster), which was higher than the inflation-adjusted value of $29,004 per EU ($1,214 per cluster) reported for Mozambique by Trotignon et al. (2017), but slightly lower than the inflation-adjusted highest value of $35,174 reported by Stelmach et al. (2019) [27,29]. Within the standard TIS data, the highest cost drivers were vehicle hire and fuel followed by per diems, which we expect to have increased since earlier studies. This result aligns with the findings of Stelmach et al. (2019), who found that per diems, vehicle hire, and fuel accounted for 85% of TIS costs [29].

A key factor impacting costs was the novel nature of the enhanced trachoma prevalence surveys. As the programmatic use of these biomarkers in both countries is not standard practice, there existed startup costs that could be avoided in the future should the activity be scaled. For example, a swab and DBS collection consultant was hired to advise the activity, with an associated cost of $143.12 per cluster in Mozambique and $131.61 per cluster in Tanzania. A consultant may not be required in the future if enhanced surveys become routine, which would result in cost savings. Additionally, the novel activity also required at least two additional days of training for the new survey team members which included ocular swab tubers and DBS technicians. As survey teams become more experienced in these methods, it is likely that less training would be required, thus reducing the associated costs. We expect that reducing training costs would be most impactful in Tanzania, where training comprised 42.9% of total costs at $1,003.52 per cluster.

Our cost-effectiveness analysis aimed to show the extent to which the increased costs of enhanced evaluation methods were commensurate with additional information provided regarding trachoma indicators within a population. In both countries, swabs and DBS were each cost-increasing and more effective than clinical grading alone, with swabs the most cost-effective in Tanzania and DBS most cost-effective in Mozambique. The enhanced methods in Tanzania resulted in greater per person incremental cost and effect than in Mozambique, likely influenced by the smaller sample size. In both countries, the combined swab and DBS method exhibited the highest costs with the same incremental effectiveness as the individual biomarkers. Since swabs were more expensive than DBS overall, this suggests that countries should select either swabs or DBS depending on their financial resources to acquire additional information regarding trachoma in their population, which will in turn aid surveillance efforts to progress toward elimination.

By applying deterministic and probabilistic sensitivity analyses to the full range of plausible ICER parameter values, our results can have applications beyond the Mozambican and Tanzanian contexts analyzed here. Tanzania and Mozambique have made substantial strides towards elimination via control programs and can serve as useful models for implementing enhanced trachoma prevalence surveys in other trachoma-endemic countries [1,3]. We found that certain context-specific costs, most notably higher per diems in Mozambique, were influential for cost-effectiveness, which can be a consideration to inform activity planning and budgeting in related settings. In the end, we found that the main factor determining the cost-effectiveness is test sensitivity, which was high for PCR and serology in this study. Lower test sensitivity could reduce cost-effectiveness in other contexts, particularly as PCR can have variable sensitivity in low-prevalence settings [5,12]. Other context-specific parameters such as acceptability and prevalence were not major factors influencing cost-effectiveness outcomes. As such, we expect our findings to be applicable to country scenarios beyond those of the study context, particularly in post-MDA settings nearing elimination.

However, an important consideration for our cost-effectiveness results is that PCR and serological tests for trachoma provide different information regarding trachoma indicators, each reflecting different stages of the natural history of the disease. Ocular swabs and PCR testing measure current *Ct* infection, while as considered here, DBS testing estimates the prevalence of antibodies against *Ct* antigens within a population. Since DBS testing detects antibody seroprevalence, likely indicating a subset of the population with previous or current infections, its assessment of the trachoma situation within a population does not directly correspond to that of PCR testing. DBS testing and modelling of age-stratified seroprevalence does allow for estimation of a measure of force of infection from a cross-sectional survey [21,37]. As such, there may be public health benefits to using both tests to monitor both current infections and transmission in a population [20,55]. For example, there could be benefits to using both methods by having one test validate the results of another, such as using PCR prevalence to confirm a decline in transmission indicated by seroprevalence [20]. As such, countries may benefit from implementing both evaluation methods to enhance the accuracy of their assessment of the trachoma situation, given they have sufficient resources. However, if a country has fewer financial resources, they will still benefit from implementing either swabs or DBS in comparison with standard grading alone.

One limitation of this study is that enhanced trachoma prevalence surveys with PCR and serological testing are not yet standard practice, and have thus not achieved the economies of scale that exist for standard trachoma prevalence surveys with clinical examination [29]. Since data on enhanced surveys is limited, we are not yet able to track costs of enhanced surveys over time or how costs might shift as the activity is scaled up. Additionally, within our data, the Tanzanian enhanced surveys were implemented as part of a sentinel site monitoring activity rather than as a TIS, with a fewer number of clusters per EU than is standard, so we expect that costs could change when applied to a larger sample size. Another consideration is that per diem and travel costs may be higher in remote areas that are harder to reach or where all the phases of survey implementation are not handled locally. As observed in our results, the categories comprising the largest share of costs varied by country, so cost-driving categories may be different in other settings. Finally, PCR testing approaches are not standardized across institutions, so differences in pooling and retesting methods could impact laboratory costs. We hope that the variation explored in our sensitivity analyses can account for some of this uncertainty inherent in future survey implementation scenarios.

Lastly, it is important to mention the limitations associated with integrating PCR and serological testing into trachoma prevalence surveys. First, the discrepancy between prevalence estimates determined by grading, PCR, and serology, each reflecting different stages of infection, presents challenges in determining which information to use for decision-making should indicators not align. WHO guidelines are based on the 5% TF threshold, and there do not yet exist guidelines surrounding the use of PCR or serology in surveys. Additionally, while PCR is highly specific, sensitivity can vary, potentially missing true positive cases [5,12,18]. Lower sensitivity could reduce the cost-effectiveness of swabs. While serological tests are sensitive and specific, *Ct* antibodies could reflect the presence of ocular or urogenital strains, so children exposed to urogenital *Ct* at birth could test positive in the absence of ocular infection [14]. This is an important consideration when interpreting serological data. Lastly, it is not clear how adding PCR and serology to trachoma prevalence surveys could impact the timeline from survey to MDA implementation given the time required for laboratory testing and interpreting results, nor how these methods could affect treatment coverage. As such, while these methods have the potential to provide information useful to trachoma control programs, more research is required to determine thresholds and develop recommendations for their programmatic use. We hope the results from this study provide useful information regarding the economic considerations of implementing such surveys.

In conclusion, we find that adding infection or serological testing to trachoma prevalence surveys using clinical grading can aid in more accurately evaluating the trachoma situation in a population in trachoma-endemic, post-MDA settings with low prevalence. For districts with persistent and recrudescent trachoma considering adding diagnostic approaches to enhance surveillance, knowing the extent of costs and information provided by these methods will be important. Our results indicated that while adding ocular swabbing and DBS testing increase the costs of a trachoma prevalence survey, the costs correspond to additional information regarding trachoma indicators that would support efforts towards elimination. The choice between swabs or DBS will depend on local capacity and the epidemiologic question of interest, being either current infection or transmission intensity. A country’s financial resources for conducting such a survey will also be the determining factor for its scalability; however, we expect costs to decrease as the survey method becomes standard and achieves economies of scale. Future research can add to the data on enhanced trachoma prevalence surveys in other countries and explore how costs change over time. As more countries reduce trachoma prevalence, enhanced methods have the potential to accelerate the pace of elimination by better targeting resources. As the first costing and cost-effectiveness study of trachoma prevalence surveys of this nature, we hope that the findings of this study can provide information useful to future surveys with *Ct* infection testing and serology in additional country contexts, facilitating progress towards the WHO’s 66-country target set for eliminating trachoma as a public health problem by 2030 [56].

## Supporting information

S1 Data Detailed cost data for Tanzania and Mozambique enhanced trachoma prevalence surveys. ***In GitHub repository:*** https://doi.org/10.5281/zenodo.14511677
(XLSX)
